# The Role of Interleukin 6 During Viral Infections

**DOI:** 10.3389/fmicb.2019.01057

**Published:** 2019-05-10

**Authors:** Lauro Velazquez-Salinas, Antonio Verdugo-Rodriguez, Luis L. Rodriguez, Manuel V. Borca

**Affiliations:** ^1^Foreign Animal Disease Research Unit, USDA/ARS Plum Island Animal Disease Center, Greenport, NY, United States; ^2^College of Veterinary Medicine and Animal Science, National Autonomous University of Mexico, Mexico City, Mexico

**Keywords:** IL-6 (interleukin 6), Th17 and Tregs cells, Virus', CD8 cytotoxic T cells +, viral persistance

Our recently published research on the characterization of vesicular stomatitis virus (VSV) pathogenesis in swine, identified a systemic upregulation of interleukin 6 (IL-6) during the acute phase of infection (Velazquez-Salinas et al., 2018). This upregulation was observed during infection with a highly virulent VSV strain, suggesting a potential association between IL-6 levels and virus virulence in pigs. In this opinion note we would like to explore in more detail the biological functions of IL-6 in different virus models, and present our perspective regarding the debatable role of IL-6 during viral infections. While several studies show the essential role of IL-6 to mount a proper immune response during some viral infections, others link this cytokine with exacerbation of viral disease. These latter findings lend support to the hypothesis that upregulation of IL-6 during certain viral infections may promote virus survival and/or exacerbation of clinical disease.

IL-6 is a pleotropic cytokine produced in response to tissue damage and infections (Tanaka et al., 2014). Multiple cell types including fibroblasts, keratinocytes, mesangial cells, vascular endothelial cells, mast cells, macrophages, dendritic cells, and T and B cells are associated with the production of this cytokine (Mauer et al., 2015). After targeting its specific receptor, IL-6 starts a cascade of signaling events mainly associated with the JAK/STAT3 activation pathway (Wang et al., 2013) promoting the transcription of multiple downstream genes associated with cellular signaling processes, including cytokines, receptors, adaptor proteins, and protein kinases (Pim-1, LDL-receptor, GADD45 beta, SOCS1, MAP3K8, SOCS3, GLUT3, HB-EGF, ICAM1, Mx1, PTP4A3, SGK, Pim-2, RHOBTB3, cAMP-GEFII, PDGF-receptor alpha, MLCK). It also controls the production of proteins implicated in regulation of gene expression (Blimp1, id-2H, MAFF, TTP, C/EBP-beta, SRY, TCF8, c-jun, junB, Bcl-3, Bcl-5, DEC1, Nmi, Stat1, eIF5, OBF-1, Oct-2, Stat3; Brocke-Heidrich et al., 2004). The number of genes regulated by IL-6 activity may explain the pleotropic nature of this interleukin. Accordingly, the biological consequences of IL-6 production have been associated with both pro- and anti-inflammatory effects (Scheller et al., 2011), highlighting IL-6's pivotal role in the activation and regulation of the immune response. Biological activities affected by production of IL-6 include: control of the differentiation of monocytes into macrophages by regulating the expression of macrophage colony-stimulating factor (Chomarat et al., 2000), increasing B-cell IgG production by regulating the expression of IL-21 (Yang et al., 2016), negative regulation of dendritic cell maturation by activation of the STAT3 signaling pathway (Park et al., 2004), as well as the promotion of the Th2 response by inhibiting Th1 polarization (Diehl and Rincon, 2002). Two different mechanisms have been described to promote the inhibition of Th1 polarization by IL-6: (1) IL-6 stimulates CD4 T cells to secrete IL-4 and direct the response to Th2, and (2) IL-6 affects the secretion of IFNγ by CD4 T cells, an essential interferon to promote Th1 polarization. A similar effect is produced in Th1 cells, where inhibition of IFNγ secretion in these cells affects CD8 T cell activation (Dienz and Rincon, 2009; Green et al., 2013).

Moreover, in combination with the transforming growing factor beta, IL-6 induces the differentiation of naïve CD4 into Th17 cells, which are important for the defense against pathogens at mucosal sites (Guglani and Khader, 2010). Also, IL-6 synergic interactions with IL-7 and IL-15 induce the differentiation and cytolytic capacity of CD8 T cells (Cox et al., 2013). Importantly, IL-6 is a potent pyrogenic cytokine, and has an essential role organizing lymphocyte trafficking to lymphoid organs during febrile events (Evans et al., 2015).

In addition to its roles modulating the host immune response, IL-6 has been implicated in the progression of several virus infectious. IL-6 is considered one of the most important cytokines during an infection, along with interleukin 1 (IL-1) and tumor necrosis factor alpha (TNF-a; Dienz and Rincon, 2009). Direct evidence supporting the importance of IL-6 during viral infections has been gathered in experimental infections using IL-6-deficient mice. Using this model, IL-6 has been shown to be essential for survival of mice infected with influenza virus by promoting optimal regulation of the T-cell response, inflammatory resolution, tissue remodeling promoting lung repair, migration and phagocytic activities of macrophages, preventing viral-induced apoptosis in lung epithelial cells, and regulation of IgG isotype switching (Lauder et al., 2013; Yang et al., 2017). Other reports have also emphasized the importance of IL-6 during virus infections. Disruption of the IL-6 gene in mice infected with vaccinia virus impaired the immune response by reducing the activity of specific cytotoxic T-cells, while murine infection with VSV impaired the production of specific IgG antibodies (Kopf et al., 1994). Additional evidence of IL-6's function during a virus infection was observed during lymphocyte choriomeningitis virus infection of mice where IL-6 and/or IL-6R activity was blocked using specific monoclonal antibodies. In this model, T helper and B-cell responses were reduced during the late stages of infection, negatively affecting viral clearance (Harker et al., 2011).

Genetically engineered rabies virus carrying the IL-6 gene in its genomic backbone has been used as an alternative model of experimentation to assess the relevance of IL-6 during viral infections (Luo et al., 2018). Normal mice infected with this virus showed a higher resistance to the viral infection compared with mice infected with the parental virus. Animals infected with the engineered virus had an increased blood-brain barrier permeability with a higher number of specific CD8-T and B-cells, increased levels of circulating neutralizing antibodies, and an intensified innate immune response in the brain as defined by up-regulation of multiple interferon-stimulated genes (ISG15, ISG20, OAS1, OAS2, and MX2).

As a warning signal during viral infections, different immune cellular pathogen recognition receptors, including toll-like receptors (TLR:2, 3, 4, 7, 8, and 9), nucleotide-binding oligomerization domain-like receptors, DNA receptors, and retinoic acid-inducible gene-1-like receptors, are able to sense a variety of pathogen-associated molecular patterns displayed by viruses (envelope glycoproteins, single and double-stranded RNA, and unmethylated CpG DNA), which stimulate transcription of IL-6 among other proinflammatory cytokines (Kawai and Akira, 2010; Tanaka et al., 2014). In this context, it has been shown that specific amino acid substitutions in a TLR-like structure in the NS4B protein of a highly virulent classical swine fever virus (CSFV) strain resulted in a completely attenuated phenotype in pigs. Infection of pigs with this mutant CSFV was characterized by the sustained accumulation of IL-6 in tonsils. Further *in vitro* experiments using exogenous IL-6 confirmed the ability of this cytokine to repress the replication of CSFV in swine peripheral blood mononuclear cells, the natural target cell during CSFV infection in pigs (Fernandez-Sainz et al., 2010).

Similarly, evidence of the antiviral effect of IL-6 was described during *in vitro* studies conducted with hepatitis B virus (HBV) where the direct ability of exogenous IL-6 to suppress the replication of this virus was described. Disruption of HBV replication was characterized by a marked decrease in the number of viral genome-containing nucleocapsids, an effect mediated in an interferon-independent manner (Kuo et al., 2009). Furthermore, IL-6 was able to block HBV infection in hepatocytes by inhibiting expression of HBV receptor in the human liver, i.e., the bile acid transporter Na (+)/taurocholate co-transporting polypeptide (Bouezzedine et al., 2015), and effectively disrupted epigenetic control of the nuclear cccDNA mini-chromosome, inhibiting HBV transcription (Palumbo et al., 2015) and the expression of hepatocyte nuclear transcription factors 1 and 4 alpha (Hosel et al., 2009).

However, experimental scientific evidence also suggests potential negative consequences that increased levels of IL-6 might have on the cellular immune response against viruses. In this context different potential mechanisms involving this cytokine might affect viral clearance, ultimately favoring the establishment of a viral persistent state in infected hosts.

First, *in-vitro* secretion of IL-6 by activated splenocytes, as a consequence of stimulation of toll like receptor 1/2 by the agonist P3C, inhibited effector CD8 T-cell responses by impairing the production of interferon gamma (IFN-γ) when compared with similarly activated and stimulated splenocytes from IL-6^−^/^−^ mice (Wu et al., 2015). Similar results were obtained providing an exogenous source of IL-6, confirming the ability of IL-6 to negatively regulate effector CD8 T-cell response after T cell activation. This inhibition was orchestrated through the STAT3 signaling pathway producing the upregulation of suppressor of cytokine signaling (SOCS3) that reduces the STAT4 phosphorylation pathway induced by IL-12, which is essential for effector CD8-T cell differentiation (Wu et al., 2015). Furthermore, *in vivo* blockage of IL-6 using a monoclonal antibody during acute infection in mice with murine leukemia virus resulted in reduced viral loads, and increased production of IFN-γ and the serine protease granzyme B (essential to produce apoptosis in target cells; Wu et al., 2015).

Second, the synergistic interaction between IL-6 and interleukin 17 (IL-17) have been associated with viral persistence and exacerbated clinical outcome during infection with Theiler's murine encephalomyelitis virus (TMEV). Genetically engineered mice carrying a human IL-6 transgene have excessive production of IL-6, leading to increased production of Th17 cells during an immune response. The IL-6 and IL-17 synergistic interaction leads to induction of anti-apoptotic molecules (Bcl-2 and Bcl-xL) inhibiting the destruction of TMEV-infected cells by virus-specific CD8+ T-cells, therefore favoring virus survival (Hou et al., 2014). Also, inhibition of apoptosis by IL-17 seems to be associated with the ability of this cytokine to block the Fas-FasL pathway (Hou et al., 2009). Interestingly, the induction of immunopathology, prevention of Th1 cells, and the inhibition of IL-2 and IFN-γ production have been mentioned as potential detrimental factors induced by Th17 cells during viral infections caused by influenza virus, Mouse hepatitis virus, hepatitis C virus, herpes simplex virus, and coxsackie virus B3 (Martinez et al., 2012).

The last potential mechanism links IL-6 with the negative co-stimulator molecules programmed cell death one (PD-1) and its ligand (PDL-1). Under normal conditions, PD-1 and PDL-1 prevent autoimmunity by inducing T-cell regulation and maintaining self-tolerance (Bardhan et al., 2016). However, during chronic viral infections, T-cell ligation of PD-1 by PDL-1, expressed on infected cells, alters immunity against viruses by preventing T-cell generation and expansion (Bardhan et al., 2016). Experimental evidence evaluating the induction of PD-1 and PDL-1 after infection with TMEV in normal and transgenic IL-6 mice showed that the excessive production of IL-6 displayed by transgenic mice after infection positively correlates with increased up-regulation of PD-1 and PDL-1 molecules in the central nervous system, and consequently with reduced CD8+ cytolytic function (Jin et al., 2013). Interestingly, PD-1 and PDL-1 up-regulation appeared to be the result of the cooperative action between IL-6 and interferon type I, with IL-6 essential to the expression of maximum levels of PDL-1 (Jin et al., 2013).

Evidence from clinical studies in humans and animals have also linked the increased systemic levels of IL-6 with the exacerbation of clinical outcomes involving viral pathogens. In this context, increased levels of IL-6 in serum has been reported in human patients chronically affected with Andes virus (Angulo et al., 2017), influenza virus (Zheng et al., 2017), HBV (Torre et al., 1994), hepatitis C virus (Spanakis et al., 2002), human immunodeficiency virus (HIV; Borges et al., 2015), Crimean-Congo hemorrhagic fever virus (Ergonul et al., 2017), and Chikungunya virus (Chirathaworn et al., 2013). Similar results have been reported in pigs and ponies infected with VSV and influenza virus, respectively, where the virulence of different strains might be positively correlated with both local and systemic detection of IL-6 (Wattrang et al., 2003; Velazquez-Salinas et al., 2018). Additionally, transcriptome analysis of persistently infected pharyngeal tissues collected from cattle with foot and mouth disease virus showed a local increase of IL-6 expression (Pacheco et al., 2015), suggesting that overexpression of IL-6 might be a possible mechanism favoring persistence of some viruses. Similarly, in HIV-infected individuals increased levels of IL-6 positively correlated with levels of residual viremia, while in ectocervical tissues, the presence of IL-6 was correlated with enhanced transcriptional levels of HIV-1 (Rollenhagen and Asin, 2011).

In conclusion, there is plentiful evidence supporting a significant role of IL-6 during viral infections. However, certain scenarios create disparity of IL-6 production that may be detrimental to the cellular immune response during viral infections. Two different hypotheses may be considered to explain the change in IL-6 production during the immune response to viral infection: (i) the increased ability of some viral strains to overcome the immune response using a variety of evasion strategies (Beachboard and Horner, 2016), and consequently up-regulate the production of IL-6 as a result of increased viral loads, and (ii) polymorphisms in the IL-6 gene promoter stimulating overexpression of IL-6 during the immune response, a fact that has been shown to correlate with HBV progression (Lan et al., 2015). This last hypothesis might explain clinical reports correlating IL-6 overexpression with exacerbation of clinical outcomes in a sub-group of individuals during an outbreak caused by a single virus strain. Interestingly, this is consistent with experimental evidence in transgenic IL-6 mice (discussed below).

Experimental evidence supports the observation that overexpression of IL-6 during the viral immune response might induce viral persistence by impairing the polarization and functionality of Th1 cells and the lytic capacity of CD8 T-cells through different mechanisms, leading to chronic infections (Figure 1). As a consequence of the constant antigen stimulation, CD8 T-cells become unresponsive and fail to develop into memory CD8 T-cells, a situation that limits viral clearance (Shin and Wherry, 2007; Bardhan et al., 2016). Increased levels of IL-6 might also exacerbate the immunopathology during chronic infections by increasing inflammation followed by cytokine secretion and cellular recruitment as described during autoimmune diseases (Srirangan and Choy, 2010). In fact, this condition of increased inflammation may be an advantage for some viruses by providing new cellular targets for subsequent viral infections (Pingen et al., 2016).

**Figure 1:**
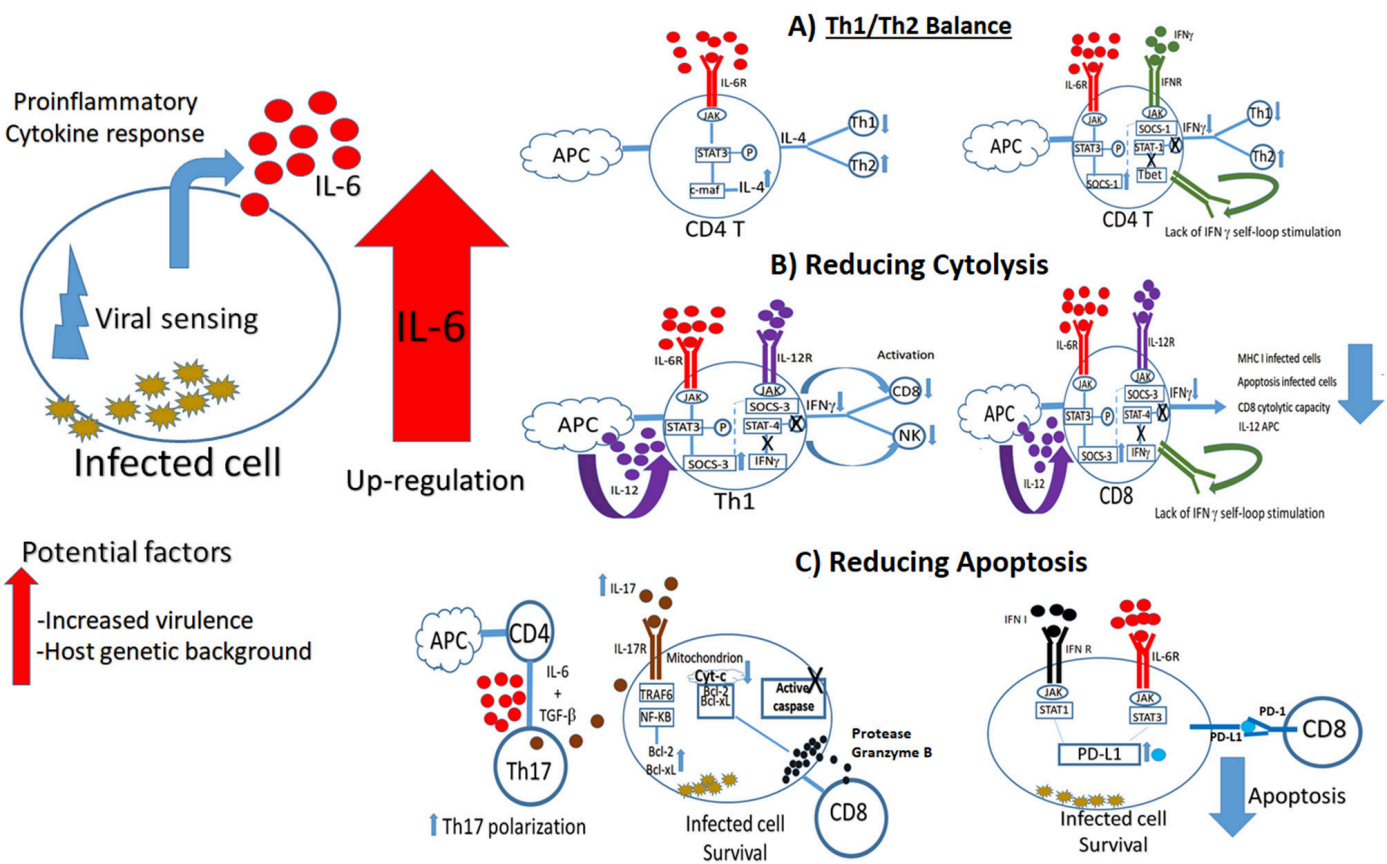
Overexpression of IL-6 and its potential negative consequences on the viral immune response. Current scientific evidence supports different scenarios where imbalance on the IL-6 production after viral infection can affect viral clearance, promoting viral persistence and chronic infections. **(A)** IL-6 might favor Th2 polarization by stimulating STAT3 pathway, and consequently increasing the production of IL-4, and the suppressor of cytokine signaling one protein (SOCS-1). SOCS-1 affects STAT 1 phosphorylation, impairing IFNγ production by decreasing IFNγ self-loop stimulation. **(B)** IL-6 might impair cytolysis by inducing the production of SOCS-3, affecting phosphorylation of STAT 4, and consequently impairing IFNγ production, an essential IFN type II interferon molecule to promote CD8 and NK cells activation. **(C)** IL-6 might promote infected cell survival by inducing apoptosis. Overexpression of this cytokine increments Th17 polarization, increasing IL-17 production in the cellular environment. IL-17 pathway induce the production of the anti-apoptotic B-cell lymphoma 2 (Bcl-2) and B-cell lymphoma extra-large (Bcl-xL) proteins, which prevent mitochondrion to produce the cytochrome complex protein (Cyt-c) after stimulation by the pro-apoptotic molecule protease Granzyme B, impairing the production of active apoptotic caspase molecules. An additional scenario includes the production of the programmed death ligand 1 protein (PD-L1) by the concerted action of IFN type I and IL-6. Matching between PD-L1 and programed death protein 1 (PD-1) switches off apoptosis mediated by CD8 T cells.

An interesting question arises regarding the potential of some viruses to selectively up-regulate IL-6 levels as a possible immune evasion strategy. Although right now there is no scientific evidence to support the causal relationship between IL-6 levels and virulence, it could open new lines of research considering the capability of other intracellular microorganisms, like *Mycobacterium tuberculosis*, to induce overexpression of IL-6 to inhibit the autophagy process in infected cells (Dutta et al., 2012)

Further work is necessary to clarify the exact role of IL-6 during virus infections and the potential role of this cytokine to be used as a biomarker of viral virulence. Additional work exploring the potential therapeutic use of blocking IL-6 or different products affected by its activity might provide insight into controlling persistent viral infections. Caution is warranted for these kinds of studies, considering the conflicting effects of this interleukin during the progression of different viral infections. It is possible that the apparent contradictory function of IL-6 may depend on diverse triggering events that can be directly linked to the characteristics inherent to each virus infection. The pleiotropism of IL-6 function might stem from different viral stimuli activating distinct patterns of effector host mechanisms and their corresponding consequences.

## Author Contributions

LV-S, AV-R, LR, and MB conceived, designed, and wrote this manuscript.

### Conflict of Interest Statement

The authors declare that the research was conducted in the absence of any commercial or financial relationships that could be construed as a potential conflict of interest.
